# Identification of the histone lysine demethylase KDM4A/JMJD2A as a novel epigenetic target in M1 macrophage polarization induced by oxidized LDL

**DOI:** 10.18632/oncotarget.17748

**Published:** 2017-05-10

**Authors:** Xue Wang, Siqing Wang, Gang Yao, Dehai Yu, Kexin Chen, Qian Tong, Long Ye, Chuan Wu, Yue Sun, Haixia Li, Dirk M. Hermann, Thorsten R. Doeppner, Fengyan Jin, Yun Dai, Jiang Wu

**Affiliations:** ^1^ Department of Neurology, the First Hospital of Jilin University, Changchun, Jilin, China; ^2^ Department of Cancer Immunology, Institute of Translational Medicine, the First Hospital of Jilin University, Changchun, Jilin, China; ^3^ Department of Neurology, the Second Hospital of Jilin University, Changchun, Jilin, China; ^4^ Cancer Center, the First Hospital of Jilin University, Changchun, Jilin, China; ^5^ Department of Immunology, Institute of Translational Medicine, the First Hospital of Jilin University, Changchun, Jilin, China; ^6^ Department of Cardiology, the First Hospital of Jilin University, Changchun, Jilin, China; ^7^ Department of Spine Surgery, the First Hospital of Jilin University, Changchun, Jilin, China; ^8^ Department of Hematology, Cancer Center, the First Hospital of Jilin University, Changchun, Jilin, China; ^9^ Laboratory of Cancer Precision Medicine, Cancer Center, the First Hospital of Jilin University, Changchun, Jilin, China; ^10^ Department of Neurology, University of Duisburg-Essen, Essen, Germany; ^11^ Department of Neurology, University of Göttingen Medical School, Göttingen, Germany

**Keywords:** KDM4A/JMJD2A, oxidized low density lipoprotein, macrophage polarization, inflammation, atherosclerosis

## Abstract

Oxidized low density lipoprotein (oxLDL) induces macrophage activation, an event essential for atherosclerosis. Emerging evidence supports that epigenetic regulation plays important roles in macrophage activation and function. However, it remains unclear which epigenetic modulator is responsible for oxLDL-induced macrophage activation. Here, we identify for the first time KDM4A (JMJD2A) as an epigenetic modifying enzyme that controls oxLDL-induced pro-inflammatory M1 polarization of macrophages. OxLDL triggered M1 polarization of murine and human macrophages, characterized by expression of iNOS and robust production of inflammatory cytokines (e.g., TNF-α, MCP-1, IL-1β). In contrast, protein level of the M2 marker Arg1 was clearly decreased after treated with oxLDL. Notably, exposure to oxLDL resulted in markedly increased expression of KDM4A in macrophages. Functionally, shRNA knockdown of KDM4A significantly impaired M1 polarization and expression of inflammatory cytokines induced by oxLDL, accompanied by increased expression of Arg1 and VEGF. However, inhibition of KDM4A by shRNA or the pan-selective KDM inhibitor JIB-04 did not affect oxLDL-mediated activation of the NF-κB and hypoxia inducible factor (HIF) pathways, and vice versa. In addition, JIB-04 induced apoptosis of macrophages in a dose-dependent manner, an event attenuated by oxLDL. Together, these findings argue that KDM4A might represent a novel epigenetic modulator that acts to direct oxLDL-induced M1 polarization of macrophages, while its up-regulation is independent of NF-κB and HIF activation, two signals critical for pro-inflammatory activation of macrophages. They also suggest that KDM4A might serve as a potential target for epigenetic therapy in prevention and treatment of inflammatory diseases such as atherosclerosis.

## INTRODUCTION

Atherosclerosis is recognized as a chronic inflammatory disease of the arterial wall, due to imbalance in lipid metabolism and uncontrolled inflammation [1]. It underlies cardiovascular (e.g., heart attack) and cerebrovascular diseases (e.g., ischemic stroke), which currently represent two leading causes of death worldwide [2]. Although the atherosclerotic process remains not fully understood, it has been well documented that macrophages play a central role throughout the pathogenesis of atherosclerosis [3]. In this context, it is a general consensus that atherosclerosis is intrinsically a persistent inflammatory process primarily involving disproportion in macrophage polarization [1]. In response to environmental stimuli, macrophages polarize into either pro-inflammatory M1 or anti-inflammatory M2 phenotypes, in a context-specific manner. The former is induced by multiple cytokines such as tumor necrosis factor-α (TNF-α), interferon-γ (IFN-γ), and granulocyte-macrophage colony-stimulating factor (GM-CSF), which in turn produces a variety of cytokines (e.g., IL-6, IL-12, IL-1β, TNF-α, MCP-1, and IL-23) to promote inflammation [4]. In contrast, the latter is induced by IL-4, IL-13, or M-CSF, which contributes to inflammation resolution via production of anti-inflammatory IL-10 and transforming growth factor-β (TGF-β) [5, 6]. Meanwhile, M2 macrophages also exhibit enhanced capability to produce vascular endothelial growth factor (VEGF) that promotes angiogenesis [7, 8]. In experimental models of atherosclerosis, pro-inflammatory M1 macrophages prevail over anti-inflammatory M2 macrophages with progression of disease [9]. In human atherosclerotic lesions, M1 macrophages are often found within the rupture-prone plaque, while M2 macrophages are usually observed in more stable plaque and away from the lipid core [9, 10]. Consistently, M1 macrophages orchestrate the whole process of atherogenesis from formation of foam cells to plaque rupture, especially via evoking and sustaining local inflammatory response [3].

Oxidized low density lipoprotein (LDL) represents a major risk factor and a core microenvironmental element of atherosclerosis. It predominantly induces polarization of macrophages towards M1 phenotype e.g., by activating toll-like receptors (TLRs) and NF-κB, thereby promoting inflammatory gene expression [11–13]. Recently, emerging findings support that epigenetic regulation may be in charge of reprogramming macrophage polarization. Histone lysine methylation, a common epigenetic post-translational modification (PTM), is reciprocally regulated by histone methyltransferases (HMTs, epigenetic “writer”) and lysine demethylases (KDMs, epigenetic “eraser”) [14]. For example, myeloid lymphoid leukemia (MLL) that catalyzes histone H3 lysine 4 trimethylation (H3K4me3) mediates M1 activation of macrophages in response to LPS or IFN-γ [15, 16]. The H3K27 demethylase JMJD3 (or KDM6B), a member of the Jumonji C-terminal domain containing enzyme family (the Jumonji family in short), contributes to polarization of macrophages towards both M1 [17–19] and M2 [20, 21], dependently upon stimuli. Of note, inhibition of HMTs prevents oxLDL-induced H3K4me3 and production of inflammatory cytokines in macrophages [15], suggesting a functional role of histone methylation in oxLDL-induced inflammation. However, no KDM has yet been identified to be involved in M1 polarization of macrophages induced by oxLDL, an event implicated in atherosclerotic pathogenesis.

Here, we report for the first time that KDM4A (also known as JMJD2A), another member of the Jumonji family essential for transcriptional regulation [22], is an epigenetic modulator responsible for M1 macrophage polarization induced by oxLDL. Whereas oxLDL sharply induced KDM4A expression, knockdown of KDM4A prevented M1 polarization and thereby dramatically impaired pro-inflammatory activity of macrophages in response to oxLDL. Moreover, up-regulation of KDM4A was independent of either NF-κB or HIF pathway activation in macropages treated with oxLDL, or *vice versa*. These findings raise a possibility that KDM4A might represent a potential target for epigentic therapy in prevention and treatment of atherosclerotic diseases and probably other inflammatory disorders as well.

## RESULTS

### OxLDL induces pro-inflammatory M1 polarization of macrophages

**Figure 1:**
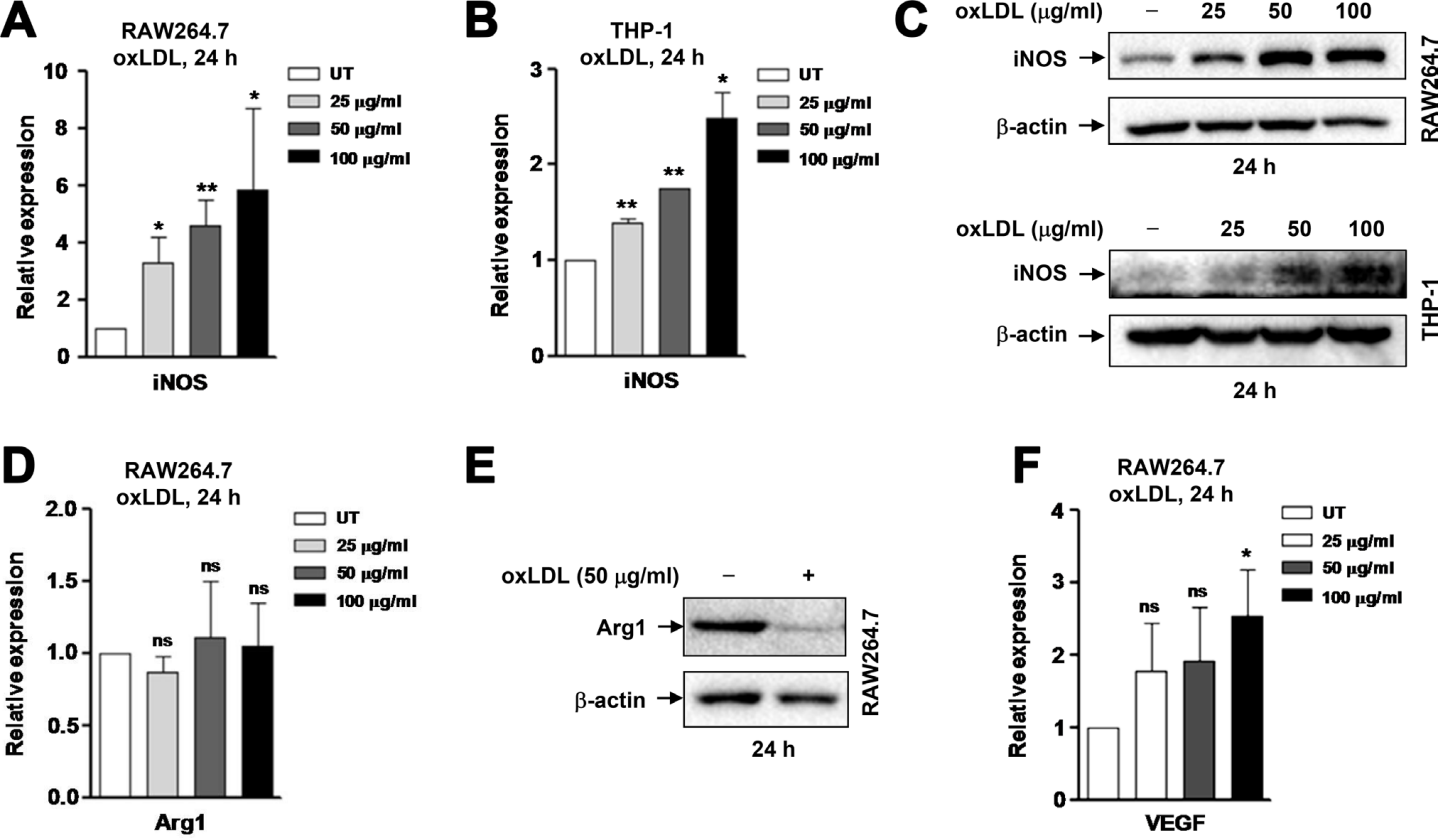
OxLDL induces M1 polarization of murine and human macrophages (**A–B**) Murine RAW264.7 cells (A) and human THP-1-derived macrophages (M0; B) were stimulated with oxLDL at 25, 50, 100 μg/ml for 24 hrs, after which expression of the M1 marker iNOS at mRNA level was analyzed by qPCR. (**C**) Alternatively, Western blot was performed to monitor the protein level of iNOS. (**D–F**) In parallel, mRNA and/or protein levels of M2-related genes, including the M2 marker Arg1 (D, E) and Vegf (F) were determined by qPCR and Western blot, respectively. Values represent the means ± SD for at least three independent experiments performed in triplicate. ^*^P < 0.05, ^**^P < 0.01, ns–not significant, compared to untreated control (UT).

It has been demonstrated earlier that oxLDL triggers M1 polarization of macrophages, thereby inducing and promoting inflammation in the pathogenesis of atherosclerosis [3]. Thus, we first verified this finding in RAW264.7 cells, a murine cell line widely used for *in vitro* studies of macrophages [23]. To this end, cells were exposed to oxLDL at concentrations of 25–100 μg/ml of 24 hrs, followed by qPCR to monitor expression of inducible nitric oxide synthase (iNOS), a typical marker for M1 macrophages [24]. As shown in Figure 1A, exposure to oxLDL resulted in a significant increase in the mRNA level of iNOS (*P* < 0.01 or 0.05, compared to untreated control). Similar results were obtained in human macrophages differentiated from THP-1 cells (by PMA; Figure 1B). Consistently, Western blot analysis also revealed that treatment with oxLDL markedly increased the protein level of iNOS in both murine and human macrophages (Figure 1C). We then examined expression of arginase 1 (Arg1), a widely-accepted marker of murine (but not human) M2 macrophages [24]. Interestingly, while qPCR did not detect any significant changes in Arg1 mRNA level after exposed to all tested concentrations of oxLDL (Figure 1D, *P* > 0.05), Western blot analysis revealed a clear decrease in the protein level of Arg1 (Figure 1E). In addition, treatment with oxLDL moderately increased expression of VEGF, a factor essential for angiogenesis [25], particularly at relatively high concentrations of oxLDL (e.g., 100 μg/ml; Figure 1F, *P* < 0.05). These findings support the notion that oxLDL induces M1 polarization of macrophages.

### OxLDL promotes production of inflammatory cytokines by macrophages

**Figure 2:**
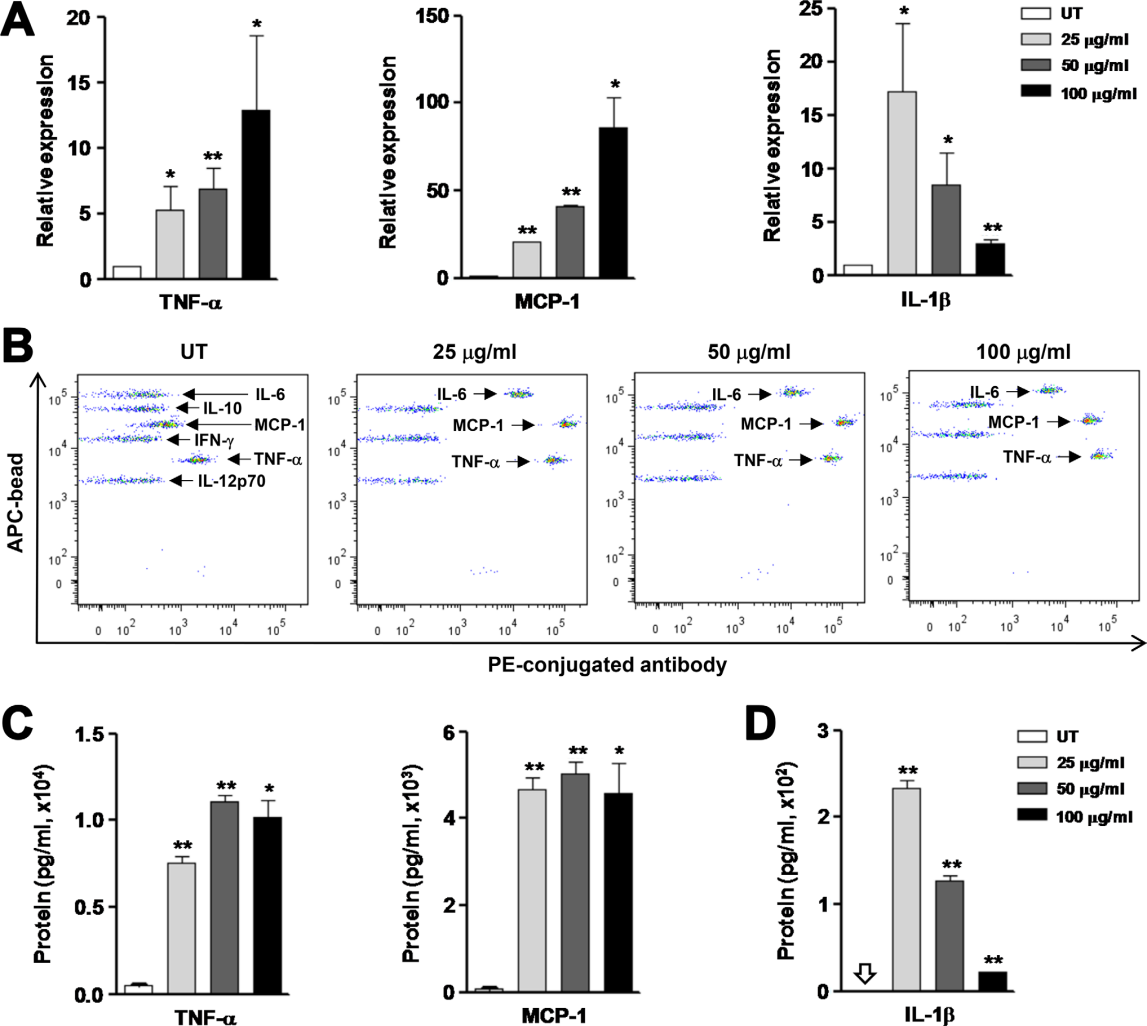
OxLDL stimulates production of inflammatory cytokines by RAW264.7 cells RAW264.7 cells were stimulated with oxLDL at 25, 50, 100 μg/ml for 24 hrs. (**A**) The mRNA levels of inflammatory cytokines, including TNF-α, MCP-1, and IL-1β, were determined by qPCR. (**B–C**) Production of the indicated cytokines was assessed by Cytometric Bead Array (CBA) using flow cytometry (B). Amount of the inflammatory cytokines TNF-α and MCP-1 measured by CBA was quantified (C). (**D**) The protein level of IL-1β in cell culture medium was determined by ELISA. Values represent the means ± SD for at least three independent experiments performed in triplicate. ^*^P < 0.05, ^**^P < 0.01, compared to untreated control (UT).

To consolidate the results that oxLDL induces pro-inflammatory M1 polarization of macrophages, we then examined whether oxLDL would also affect expression of M1-related inflammatory genes. Indeed, exposure of RAW264.7 cells to oxLDL at the same concentrations as above (Figure 1) for 24 hrs led to a significant increase in the mRNA levels of inflammatory genes, including TNF-α, IL-1β, MCP-1 (Figure 2A, *P* < 0.01 or 0.05, compared to untreated control), as well as IL-6 and to a lesser extent IFN-γ (*P* < 0.01 for IL-6, *P* > 0.05 for IFN-γ; Supplementary Figure 1A). Induction of M1 gene expression by oxLDL was also seen in THP-1-derived macrophages (e.g., TNF-α, *P* > 0.05 for 25 μg/ml oxLDL, *P* < 0.05 for 50 and 100 μg/ml oxLDL; Supplementary Figure 1B). However, we did not observe any significant changes in expression of M2-related anti-inflammatory genes such as IL-10 and TGF-β in RAW264.7 cells treated with oxLDL (*P* > 0.05 for each case; Supplementary Figure 1C). Further, the protein levels of M1 cytokines in culture medium were determined by flow cytometry using a Cytometric Bead Array (CBA) kit that is designed for detecting up to six soluble factors (including TNF-α, MCP-1, IL-6, IFN-γ, IL-10, and IL-12p70) [26]. As shown in Figure 2B, treatment of RAW264.7 cells with oxLDL resulted in a sharp increase (right shift) in the protein levels of TNF-α, MCP-1, and IL-6. However, oxLDL exposure did not affect the levels of M2 cytokines such as IL-10 (Figure 2B), consistent with no changes in the mRNA levels of these genes (Supplementary Figure 1C). Interestingly, treatment with oxLDL induced neither production of another M1 cytokine IL-12p70 (Figure 2B) nor gene expression of IL-12p40 and p35 (data not shown), two subunits of the active cytokine heterodimeric IL-12p70. The quantified results for M1 cytokine protein levels in supernatant revealed robust production of TNF-α (> 7 ng/ml), MCP-1 (> 4 ng/ml), and IL-6 (∼200 pg/ml to > 4 ng/ml, data not shown) by macrophages after stimulated by oxLDL (Figure 2C, *P* < 0.01 or 0.05). Because IL-1β is not covered by this CBA kit, its concentration in supernatant was determined by an ELISA assay. As shown in Figure 2D, exposure to oxLDL also significantly increased production of IL-1β by macrophages (*P* < 0.01). Together, these findings indicate that oxLDL induces macrophages to produce a large amount of inflammatory cytokines, arguing that oxLDL triggers macrophage M1 polarization to promote inflammation in atherosclerosis.

### OxLDL up-regulates expression of the lysine demethylase KDM4A in macrophages

**Figure 3:**
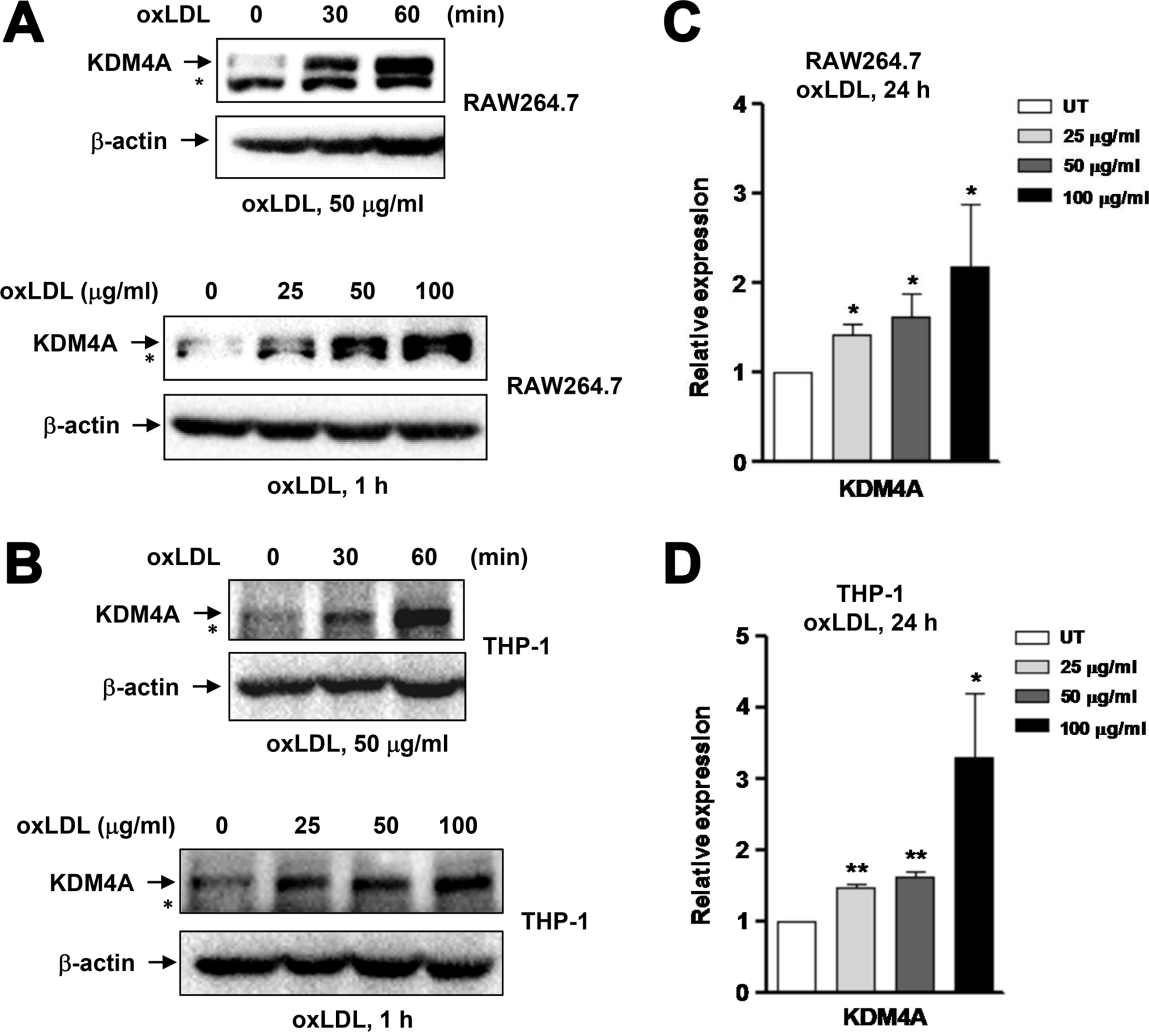
Exposure to oxLDL results in up-regulation of KDM4A in macrophages (**A–B**) RAW264.7 cells (A) and human THP-1-derived macrophages (B) were incubated with oxLDL (50 μg/ml) for 30–60 minutes (upper panels), or at concentrations of 25, 50, 100 μg/ml for 60 minutes (lower panels), after which Western blot analysis was performed to monitor the protein level of KDM4A. ^*^undefined band. (**C–D**) Alternatively, the mRNA level of KDM4A was determined by qPCR in RAW264.7 cells (C) and human THP-1-derived macrophages (D) after exposed to oxLDL at 25, 50, 100 μg/ml for 24 hrs. Values represent the means ± SD for at least three independent experiments performed in triplicate. ^*^P < 0.05, ^**^P < 0.01, compared to untreated control (UT).

Increasing evidence supports a concept that epigenetic regulation governs reprogramming of macrophage polarization. Among histone PTMs, lysine methylation is reciprocally regulated by HMTs and KDMs [14]. For the latter, JMJD3 is the only one lysine demethylase identified so far that is functionally involved in macrophage polarization. However, JMJD3 seems to be able to mediate macrophage polarization towards both M1 and M2, likely in a context-specific manner [17, 21]. As another member of the Jumonji family, KDM4A has been widely studied in cancer [27], while not yet in either macrophages or inflammatory diseases including atherosclerosis. Therefore, we sought to examine whether KDM4A would also play a role in macrophage polarization, particularly induced by oxLDL. Notably, exposure of RAW264.7 cells to 25–100 μg/ml oxLDL induced expression of KDM4A rapidly (Figure 3A, upper panels) and in a dose-dependent manner (lower panels). To validate this finding in human macrophages, THP-1 cells were differentiated to macrophages (M0) by incubation with 30 ng/ml PMA for 48 hrs, a well-established approach to prepare macrophages from human origin [28]. After washed out PMA for 24 hours, the macrophages were then treated with oxLDL. Virtually identical to the results obtained from murine macrophages (Figure 3A), exposure to the same concentrations of oxLDL (i.e., 25– 100 μg/ml) for a short interval (e.g., ≤ 1 hour) also led to a marked increase in KDM4A expression in THP-1-derived human macrophages (Figure 3B). To test whether KDM4A expression would be sustained, qPCR was performed to detect expression of KDM4A in macrophages after prolonged incubation with oxLDL. After exposed to oxLDL for 24 hrs, the mRNA level of KDM4A remained significantly increased in both RAW264.7 cells (Figure 3C, *P* < 0.05, compared to untreated control) and THP-1-derived macrophages (Figure 3D, *P* < 0.01 or 0.05). Therefore, these findings indicate that oxLDL induces KDM4A expression in macrophages.

### KDM4A knockdown prevents oxLDL-induced M1 polarization of macrophages

**Figure 4:**
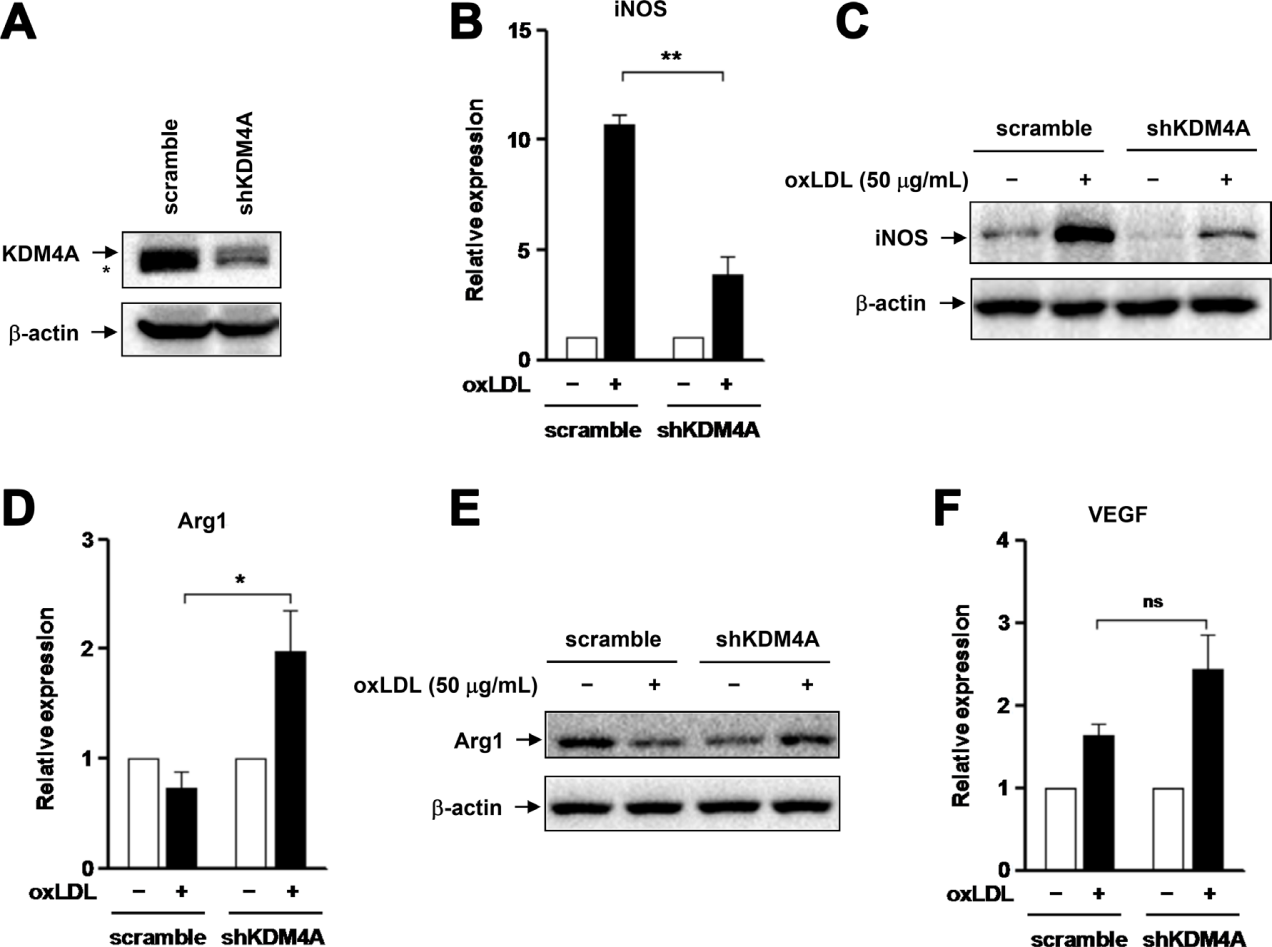
Knockdown of KDM4A attenuates oxLDL-induced M1 polarization of RAW264.7 cells (**A**) RAW264.7 cells were transiently transfected with pGreenpuro-shRNA constructs specifically targeting KDM4A (shKDM4A) or scrambled sequence as control. Western blot analysis demonstrates down-regulation of KDM4A by shRNA, compared to scramble control. (**B–C**) RAW264.7 cells transfected with shKDM4A and scramble control shRNA were exposed to 50 μg/ml oxLDL for 24 hrs, after which expression of iNOS at mRNA (B) or protein level (C) was examined by qPCR and Western blot, respectively. (**D–F**) In parallel, the mRNA and/or protein levels of Arg1 (D, E) and Vegf (F) were measured by qPCR and Western blot, respectively. Values represent the means ± SD for at least three independent experiments performed in triplicate. ^*^P < 0.05, ^**^P < 0.01, ns–not significant, compared to scramble control.

We next examined whether KDM4A plays a functional role in oxLDL-induced M1 polarization of macrophages. To this end, KDM4A was knocked down using an shRNA specifically targeting KDM4A in RAW264.7 cells, manifested by marked down-regulation of KDM4A expression at protein level, compared to an shRNA against a scrambled sequence as negative control (Figure 4A). Similar results were obtained using another independent shRNA targeting KDM4A (Supplementary Figure 2A). Transfected cells were then exposed to 50 μg/ml oxLDL for 24 hrs, after which the mRNA and protein levels of iNOS were monitored by qPCR and Western blot analysis, respectively. Notably, knockdown of KDM4A sharply diminished oxLDL-induced expression of iNOS at both mRNA (Figure 4B, *P* < 0.01, compared to negative control) and protein levels (Figure 4C). In contrast, oxLDL markedly increased expression of Arg1 at mRNA level in cells with KDM4A knockdown (Figure 4D, *P* < 0.05, compared to negative control), but did not affect the mRNA level of Arg1 in negative control cells, consistent with the observation in untransfected RAW264.7 cells (Figure 1C). Moreover, Western blot analysis revealed that treatment with oxLDL led to a marked increase in the protein level of Arg1 in cells with KDM4A knockdown, while clearly attenuated expression of Arg1 protein in negative control cells (Figure 4E), analogous to the result obtained from untransfected cells (Figure 1D). Interestingly, compared to negative control, KDM4A knockdown resulted in a noticeable reduction in the basal level of Arg1 protein (Figure 4E), via a mechanism that remains to be defined. Last, oxLDL moderately up-regulated VEGF expression in negative control cells (Figure 4F), as observed in untransfected cells (Figure 1E). This event was clearly, although not statistically significantly, enhanced by KDM4A knockdown (Figure 4F), consistent with the previous observation that M2 macrophages often display an increased capability to produce VEGF [29]. Therefore, these findings indicate that KDM4A plays a functional role in M1 polarization of macrophages induced by oxLDL, while targeting KDM4A might redirect macrophage polarization from M1 to M2 phenotype.

### KDM4A knockdown prevents expression of inflammatory genes in macrophages exposed to oxLDL

**Figure 5:**
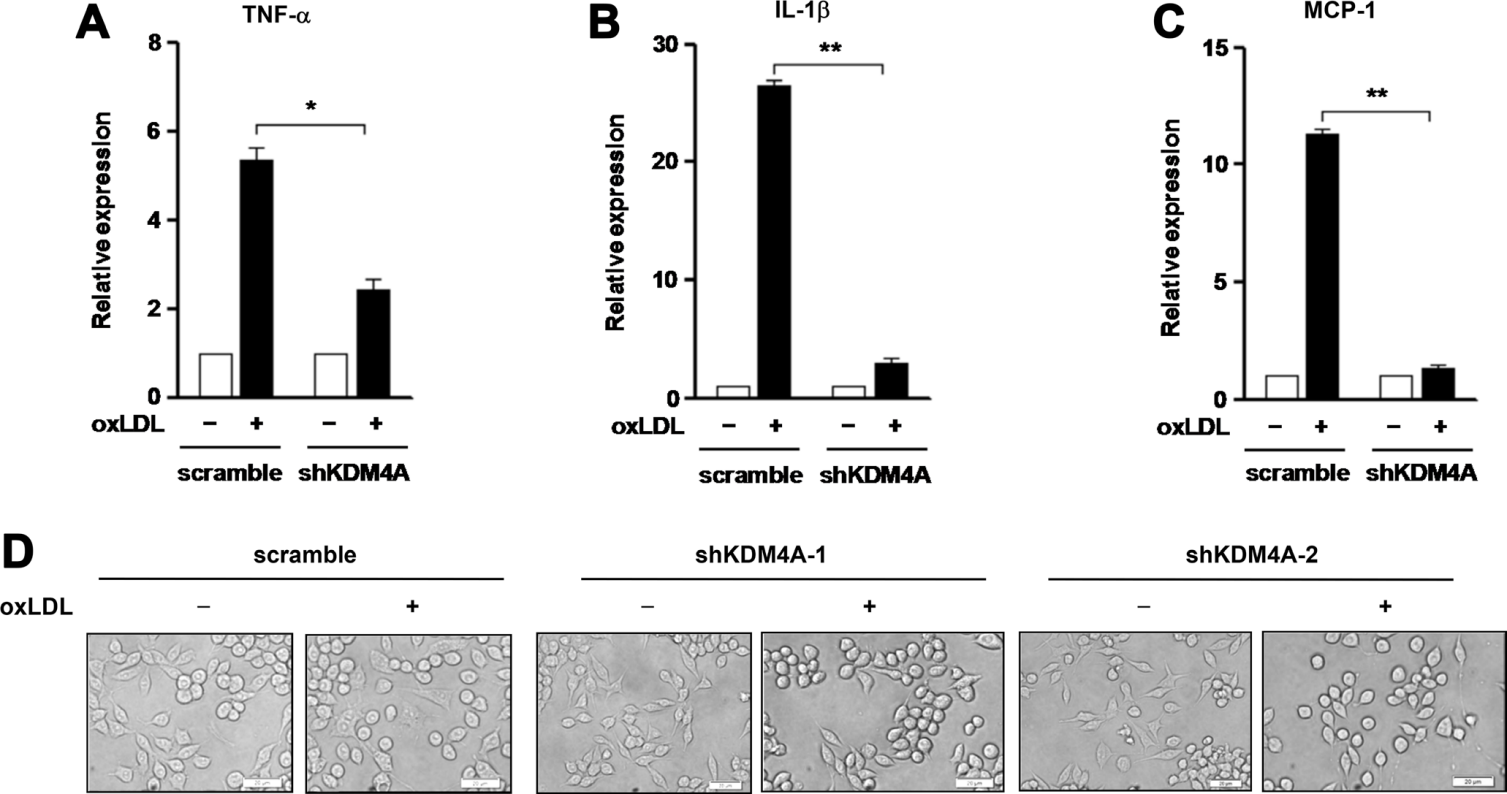
Knockdown of KDM4A prevents expression of inflammatory genes induced by oxLDL in RAW264.7 cells (**A–C**) RAW264.7 cells transfected with shKDM4A and scramble control vector were exposed to 50 μg/ml oxLDL for 24 hrs, after which mRNA levels of TNF-α (A), IL-1β (B), and MCP-1 (C) were determined by qPCR. (**D**) Representative microscopic images were captured under converted light microscopy, to observe morphological changes of cells after exposure to 50 μg/ml oxLDL for 24 hrs. Two constructs designated shKDM4A-1 and –2 targeting different sequences of the KDM4A gene were used to confirm the morphological observations. Values represent the means ± SD for at least three independent experiments performed in triplicate. ^*^P < 0.05, ^**^P < 0.01, compared to untreated control.

We further tested whether KDM4A deficiency would also affect oxLDL-induced production of M1 cytokines by macrophages. To this end, qPCR was performed to monitor expression of three representative M1 inflammatory genes, including TNF-α, IL-1β, and MCP-1. Consistent with defect of M1 polarization (Figure 4B and 4C), KDM4A knockdown dramatically reduced oxLDL-induced expression of TNF-α (Figure 5A) and IL-1β (Figure 5B), and virtually completely eliminated expression of MCP-1 (Figure 5C; for 5A-5C, *P* < 0.01 or 0.05, compared to those for negative control). In addition, after treated with oxLDL for 24 hrs, RAW264.7 cells with KDM4A knockdown exhibited the morphology of inactive macrophages, featured by emboss-like round shape [30], a phenomenon not observed in negative control cells (Figure 5D; shKDM4A-1 and -2, two independent shRNAs). Together, these findings argue strongly that KDM4A is an epigenetic modulator that controls pro-inflammatory M1 polarization and activation of macrophages in response to oxLDL, which might therefore imply a novel mechanism for epigenetic reprogramming of macrophages as well as a potential therapeutic target for the treatment of inflammatory diseases such as atherosclerosis.

### OxLDL-induced KDM4A expression is independent of NF-κB and HIF activation

**Figure 6:**
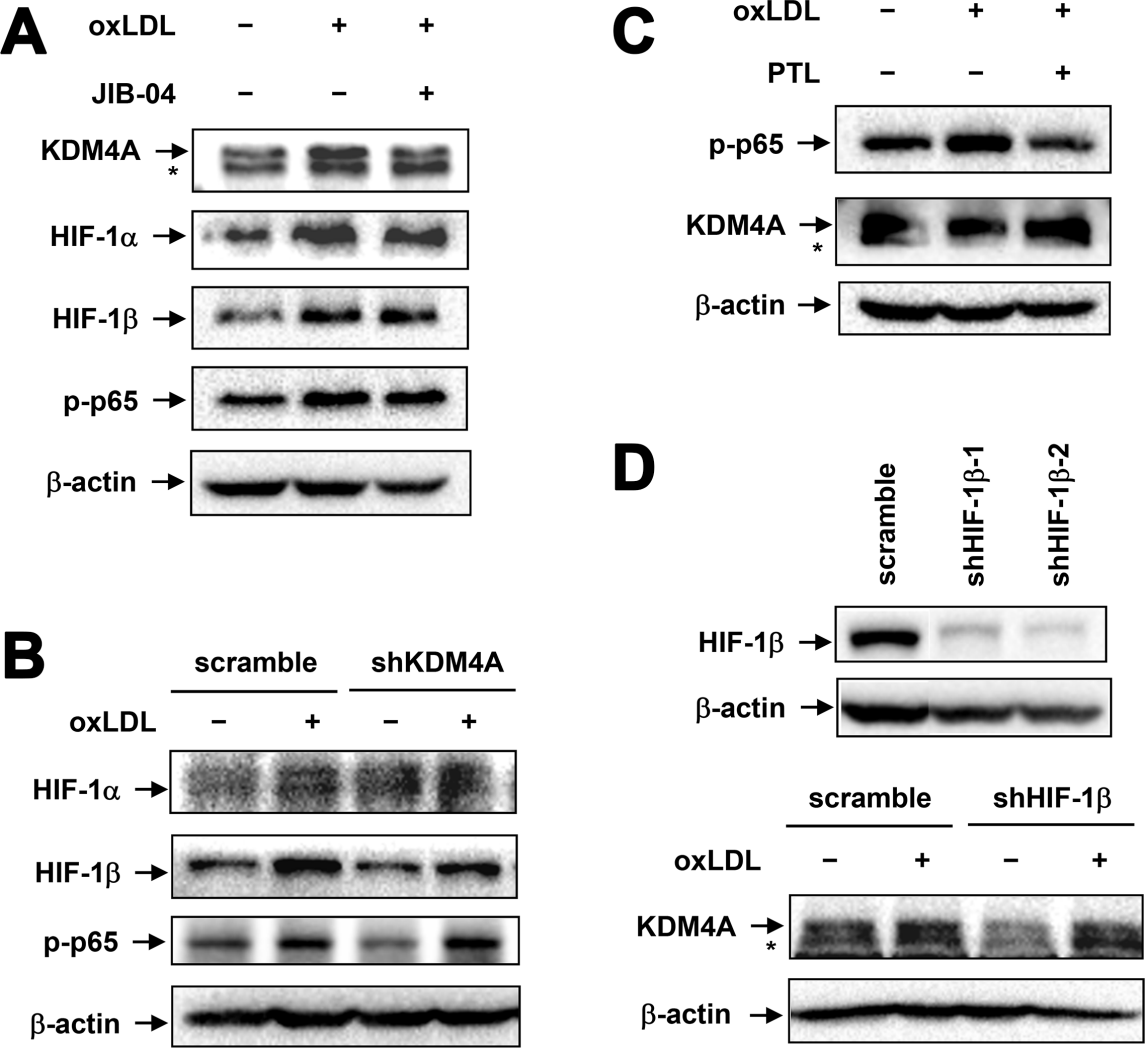
Up-regulation of KDM4A is independent of NF-κB and HIF activation in RAW264.7 cells exposed to oxLDL (**A**) RAW264.7 cells were exposed for 1 hr to 50 μg/ml oxLDL with or without pre-treatment with the JMJD inhibitor JIB-04 for 4 hrs, after which Western blot analysis was carried out to monitor expression of the indicated proteins. (**B**) RAW264.7 cells transfected with shKDM4A and scramble control shRNA were exposed to 50 μg/ml oxLDL for 1 hr, after which expression of HIF-1α, -1β, and phosphorylated p65 (S536) was monitored by Western blot analysis. (**C**) RAW264.7 cells were exposed for 1 hr to 50 μg/ml oxLDL with or without pre-treatment with the IKK inhibitor parthenolide (PTL) for 2 hrs, after which expression of phosphorylated p65 and KDM4A was assessed by Western blot analysis. (**D**) RAW264.7 cells were transiently transfected with pGreenpuro-shRNA constructs specifically targeting HIF-1β (shHIF-1β-1 and -2) or scrambled sequence as control. Down-regulation of HIF-1β was validated by Western blot analysis (upper panel). Cells were then exposed to 50 μg/ml oxLDL for 1 hr, after which expression of KDM4A was monitored by Western blot analysis.

It is well documented that the NF-κB and HIF pathways are activated and play a crucial role in macrophage activation, including M1 polarization [31, 32]. In this context, we examined whether oxLDL-induced expression of KDM4A would be associated with activation of these two signaling pathways. As shown in Figure 6A, exposure of RAW264.7 cells to 50 μg/ml oxLDL for 1 hr remarkably induced expression of HIF-1α and HIF-1β, reflecting activation of the HIF pathway [33], as well as increased S536 phosphorylation of p65 (RelA) that is primarily catalyzed by IKKβ, indicating activation of the canonical NF-κB pathway [34]. Notably, pretreatment for 4 hrs with 1 μM JIB-04, a pan-selective inhibitor of KDMs (including KDM4A) [35], clearly diminished oxLDL-mediated up-regulation of KDM4A, while it did not affect either expression of HIFs (e.g., HIF-1α and HIF-1β) or phosphorylation of p65 induced by oxLDL (Figure 6A). In addition, JIB-04 had also no effect on oxLDL-induced S32/S36 phosphorylation of IκBα (Supplementary Figure 2B), another direct downstream target of IKKβ. Similar results were obtained in cells with KDM4A knockdown (Figure 6B). Conversely, parthenolide, an IKK inhibitor [36], blocked p65 phosphorylation induced by oxLDL. However, pre-treatment for 2 hrs with parthenolide failed to affect KDM4A up-regulation after exposed to oxLDL (Figure 6C) or lipopolysaccharide (LPS; Supplementary Figure 2C), a stimulus known to induce M1 phenotype via NF-κB activation [23]. Moreover, HIF-1β knockdown by shRNA (upper panel) also did not alter oxLDL-induced expression of KDM4A (lower panel, Figure 6D). Therefore, these findings indicate that oxLDL induces expression of KDM4A via a process independent of NF-κB and HIF pathway activation, suggesting that KDM4A-mediated reprogramming of macrophage polarization might involve an alternative mechanism.

### KDM inhibition induces apoptosis of macrophages, an event prevented by oxLDL

**Figure 7:**
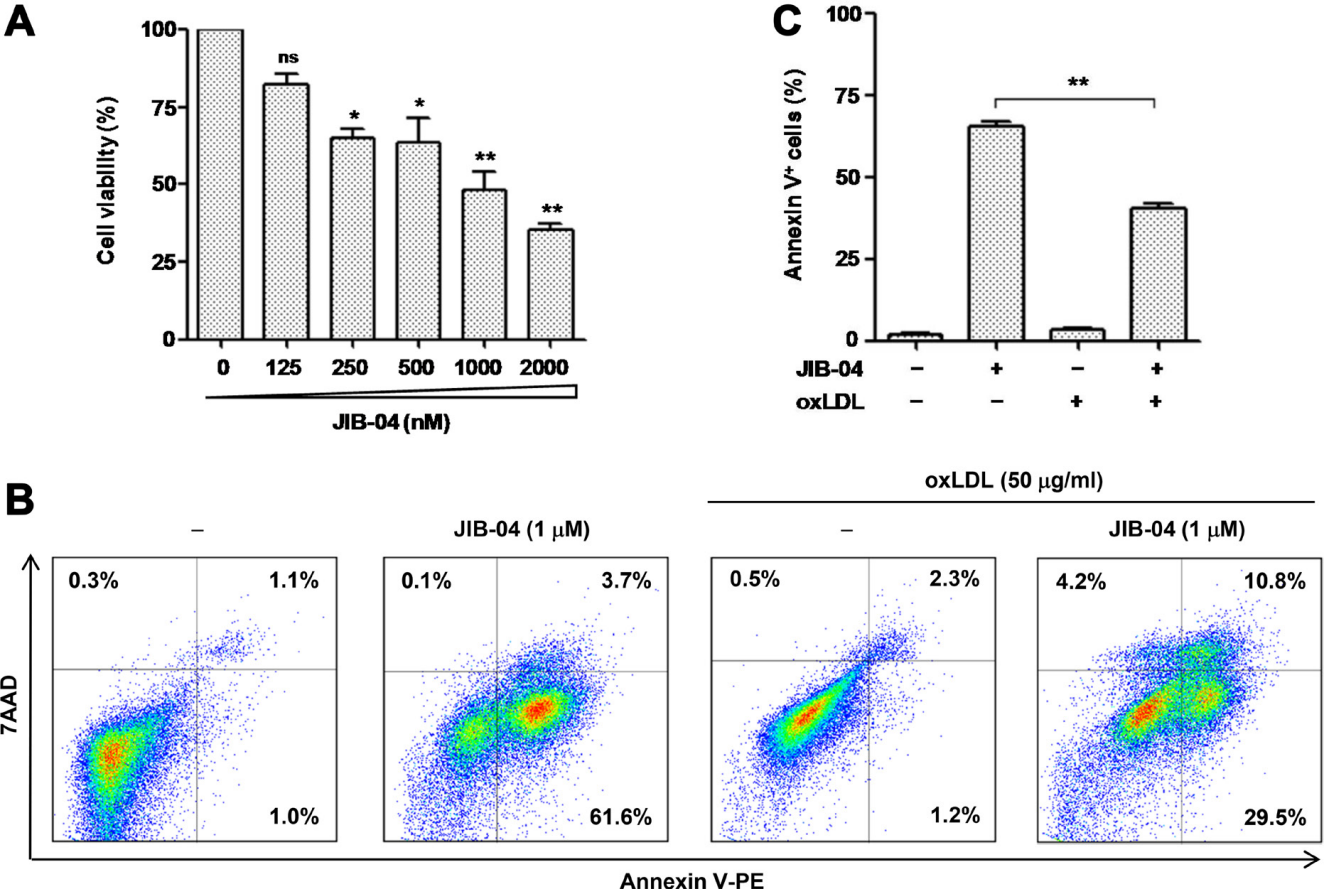
The JMJD inhibitor JIB-04 induces apoptosis of RAW264.7 cells, an event attenuated by oxLDL (**A**) RAW264.7 cells were treated with a series of concentrations of JIB-04 for 24 hrs, after which cell viability was analyzed using a CCK8 kit. (**B–C**) RAW264.7 cells were exposed to 50 μg/ml oxLDL in the presence or absence of 1 μM JIB-04 for 24 hrs, after which cells were double stained with Annexin V-PE and 7AAD, followed by flow cytometry to determine the percentage of apoptotic cells. Representative data of flow cytometry were shown to analyze early (Annexin V^+^/7AAD^−^) and late (Annexin V^+^/7AAD^+^) apoptosis (B). Values represent the means ± SD for at least three independent experiments performed in triplicate. ^**^P < 0.01, compared to untreated control.

Last, it has been reported that KDM4A depletion induces apoptosis in tumor cells [37]. We thus examined whether KDM4A inhibition would trigger apoptosis of macrophages, an event involved in atherogenesis [38]. To this end, RAW264.7 cells were treated with a series of concentrations of JIB-04, after which cell viability was monitored using a CCK-8 assay. As shown in Figure 7A, exposure to JIB-04 resulted in a dose-dependent decline in cell viability of macrophages (*P* < 0.01 or 0.05, compared to untreated control). Further, treatment with 1 μM JIB-04 for 24 hrs induced robust apoptosis of macrophages, reflected by increased percentage of Annexin V-positive cells (Figure 7B and 7C). Interestingly, the lethality of JIB-04 was significantly attenuated by co-administration of 50 μg/ml oxLDL. Therefore, these findings suggest that KDM4A might also play a pro-survival role in macrophages, while oxLDL could neutralize the lethal action of KDM inhibition via up-regulation of KDM4A.

## DISCUSSION

A growing body of research has unveiled the roles of epigenetic regulation in a multitude of macrophage functions in a variety of inflammatory diseases, including atherosclerosis that has a major impact on human health. Histone lysine methylation represents a key epigenetic code for gene expression, which has recently been linked to macrophage polarization towards either pro-inflammatory M1 or anti-inflammatory M2 phenotype [39]. However, it remains unclear which histone epigenetic modifying enzyme is responsible for reprogramming M1 polarization of macrophages induced by oxLDL, an event essential for the pathogenesis of atherosclerosis [1, 24, 40]. In the present study, we identified KDM4A as a histone lysine demethylase that controls oxLDL-induced M1 polarization of macrophages and production of inflammatory cytokines and chemokines. It was found that exposure to oxLDL resulted in a rapid and persistent increase in expression of KDM4A in both murine and human macrophages. Of note, knockdown of KDM4A by shRNA sharply attenuated oxLDL-induced M1 polarization of macrophages and production of inflammatory factors (e.g., TNF-α, IL-1β, IL-6, MCP-1). Interestingly, oxLDL-mediated up-regulation of KDM4A was independent of activation of the NF-κB and HIF signaling pathways that are known to be involved in macrophage activation under diverse inflammatory conditions including atherosclerosis. Therefore, these findings argue that KDM4A is an important epigenetic modulator that governs reprogramming of macrophage M1 polarization in response to oxLDL, and therefore might represent a potential novel therapeutic target for epigenetic therapy of atherosclerotic diseases. They also provide a new insight into the mechanism underlying uncontrolled inflammation mediated by macrophages in atherosclerosis.

Macrophages are highly plastic. They can transform from one phenotype to another dependently upon different stimuli in microenvironment [40]. Following endothelial activation and/or dysfunction, circulating LDL invades the endothelium and deposits in the vascular wall where it is oxidized to initiate atherosclerosis, an event that is often promoted by hyperlipidemia [3]. As a major risk factor of atherosclerosis [41], oxLDL is a potent pro-inflammatory factor that recruits monocytes from peripheral blood to early atherosclerotic lesion where they differentiate to macrophages and then become foam cells via uptake of oxLDL, a hallmark of atherosclerosis [3]. Notably, oxLDL also activates macrophages to produce a large amount of inflammatory factors to trigger inflammation [15], a critical event that drives atherosclerotic pathogenesis [1]. Consistent with these earlier findings, we observed that *in vitro* exposure to oxLDL induced macrophage polarization towards pro-inflammatory M1 phenotype featured by expression of iNOS, a classic M1 marker [24]. Moreover, this event was associated with increased gene expression and robust production of inflammatory cytokines (e.g., TNF-α, IL-1β, IL-6) and chemokines (e.g., MCP-1). In contrast, treatment with oxLDL markedly reduced the protein level of Arg1, a marker of murine M2 macrophages [29], although it did not affect its mRNA level. Interestingly, it has been found that treatment of M2-polarized macrophages with oxLDL also promotes production of M1-related cytokines (e.g., IL-6, IL-8, and MCP-1), while suppresses secretion of anti-inflammatory M2 cytokines such as IL-10 [42]. Thus, it is possible that in addition to promotion of M1 polarization, oxLDL might directly induce production of inflammatory cytokines by macrophages. Moreover, oxidized phospholipids in oxLDL can transform macrophages to another phenotype (designated Mox), which like M1 macrophages, display limited capacity of phagocytosis and migration but express inflammatory genes (e.g., IL-1β and COX-2) [43]. Nevertheless, the present study provides evidence supporting a notion that oxLDL induces pro-inflammatory M1 polarization of macrophages, which might contribute to persistent inflammation without resolution within atherosclerotic lesions.

DNA methylation and histone PTMs represent two major mechanisms for epigenetic regulation of gene expression without altering the DNA sequence. Lysine methylation and acetylation are widely accepted as the most important histone PTMs to determine accessibility of transcription factors to the promoter and enhancer regions of target genes via chromatin remodeling. While these epigenetic codes are written by HMTs or histone acetyltransferases (HATs), they are erased by KDMs or histone deacetylase (HDACs), respectively [14, 44]. Recently, emerging evidence supports a critical role of these histone modifying enzymes in regulation of macrophage activation and function, which thereby provides a rationale for targeting these enzymes as a potential epigenetic therapy in inflammatory diseases such as atherosclerosis. For example, LPS-induced expression of M1 genes (e.g., iNOS, IL-6, TNF-α) is severely impaired in bone marrow-derived macrophages of HDAC3-deficient mice [45]. Deletion of HDAC3 directs macrophages towards a state similar to IL-4-induced alternative phenotype with enhanced expression of M2 genes such as Arg1, Retnla, Clec7a, and chi3l3 [46]. In this context, targeting HDAC3 in macrophages stabilizes atherosclerotic lesions [47]. HDAC7 has also been reported to promote toll-like receptor 4 (TLR4)-dependent expression of inflammatory genes in macrophages [48]. A recent analysis of the genome-wide association studies (GWAS) has discovered a new association of large vessel ischemic stroke with a genetic variant of HDAC9 [49]. HDAC9 expression is up-regulated during macrophage differentiation, which in turn promotes M2 polarization but suppresses expression of M1 inflammatory genes via chromatin remodeling [50]. Further, deficiency of HDAC9 attenuates atherosclerosis in ApoE^−/−^ mice [51]. In addition, the high level of HDAC9 in differentiated macrophages is maintained by the DNA methyltransferase DNMT3a [52]. In the case of histone methylation, JMJD3 is the only one KDM identified so far to be functionally involved in regulation of macrophage polarization and function. In JMJD3-deficient macrophages, LPS- or SAA-induced expression of M1 genes is largely diminished [17, 18]. Similarly, inhibition of H3K27me3 demethylases (including JMJD3) by a selective inhibitor prevents LPS-induced production of inflammatory cytokines by human primary macrophages [19]. However, the classic M2 stimulus IL-4 also induces expression of JMJD3 in macrophages [20]. Moreover, JMJD3 mediates M2 polarization of macrophages in response to helminth infection, in association with transcriptional activation of IRF4 expression due to specific demethylation of H3K27me3 at the promoter region of IRF4 gene [21]. In contrast, GM-CSF, a classic M1 stimulus, up-regulates IRF4 expression by enhancing JMJD3 demethylase activity [53]. KDM4A (JMJD2A) is another member of the Jumonji family, which demethylates H3K9me3, H3K36me3, and H1.4K26me3 [27]. Although KDM4A is one of the most studied KDMs in cancer, neither its role in regulation of macrophage function and inflammation nor its relationship with oxLDL and atherosclerosis has been explored before. In the present study, we identified KDM4A as a novel lysine demethylase functionally involved in reprogramming of macrophage polarization. Unlike JMJD3 that mediates both M1 and M2 polarization of macrophages, KDM4A appears to primarily direct M1 polarization, at least in the present setting. While it remains to be defined whether JMJD3 also contributes to inflammation involved in atherosclerosis, KDM4A is the first histone lysine demethylase, to the best of our knowledge, identified to be involved in regulation of macrophage polarization and function in response to oxLDL, an event particularly implicated in atherosclerosis. A possibility thus arises that KDM4A might participate in uncontrolled inflammation, which is required for initiation and progression of atherosclerosis, by governing oxLDL-induced pro-inflammatory M1 polarization of macrophages. Of note, targeting KDM4A not only prevented oxLDL-induced M1 polarization of macrophages, but also redirected them towards anti-inflammatory M2 phenotype that is known to promote inflammation resolution in atherosclerosis. Therefore, in conjunction with the earlier findings that inhibition of histone lysine methylation blocks oxLDL-induced production of inflammatory cytokines by macrophages [15], the present observations argue that KDM4A might serve as a novel target for the emerging epigenetic therapy of atherosclerotic diseases.

It has been well documented that the NF-κB and HIF pathways as well as their interactions play a crucial role in transcriptional regulation of macrophage activation and function. Whereas both HIF-1 and NF-κB are able to promote expression of inflammatory genes in macrophages [31, 32], HIF-1α activates NF-κB that in turn controls HIF-1α transcription, thereby linking hypoxic response to innate immunity and inflammation [54]. There also exist numerous cross-talks between the transcriptional and epigenetic mechanisms in regulation of macrophage function. For example, NF-κB activation induces JMJD3 expression in response to inflammatory stimuli in primary mouse macrophages [55]. Conversely, JMJD3 transcriptionally up-regulates multiple targets of NF-κB signaling [56]. Moreover, JMJD3 interacts with NF-κB to promote expression of inflammatory genes in keratinocytes during wound healing [57]. It is also noteworthy that multiple members of the Jumonji family are transcriptional targets of HIFs. For example, hypoxia induces JMJD3 via HIF-1α and HIF-1β, while HIF-1α activation increases JMJD3 expression even under normoxia [58]. In addition, HIF-1α also up-regulates JMJD1A, JMJD2B, and JMJD2C [59, 60]. Thus, a question arises whether expression of KDM4A in macrophages exposed to oxLDL would be linked to activation of the NF-κB or HIF pathways. However, the present results indicate that neither inhibition of NF-κB activation nor knockdown of HIF-1β, a constitutively stable subunit of active HIF heterodimer with HIF-1α, was able to alter oxLDL-induced expression of KDM4A. Conversely, knockdown of KDM4A failed to affect activation of the NF-κB and HIF pathways in response to oxLDL. Therefore, these findings indicate that KDM4A up-regulation in macrophages after exposed to oxLDL is a separate event from NF-κB and HIF activation, suggesting an alternative signaling cascade involved in regulation of macrophage activation and inflammation in the pathogenesis of atherosclerosis.

KDM4A is involved in regulation of apoptosis in tumor cells [35, 61]. In the present study, we observed that the pan-selective JMJD inhibitor JIB-04 induced robust apoptosis of macrophages, implying a functional role of the JMJD family in macrophage survival. Interestingly, co-administration of oxLDL partially but significantly prevented apoptosis induced by JIB-04. Although the mechanism for this phenomenon is unknown, one possible explanation is that KDM4A up-regulated by oxLDL might play a pro-survival role by neutralizing the lethal action of JMJD inhibition in macrophages. However, as JIB-04 inhibits multiple KDMs (including KDM4A) [41], it remains to be defined which KDM inhibition accounts for the lethality of JIB-04 against macrophages.

In conclusion, the present findings indicate that the Jumonji demethylase KDM4A (or JMJD2A) is a novel epigenetic modulator that mediates pro-inflammatory M1 polarization of macrophages in response to oxLDL. They also argue that oxLDL-induced up-regulation of KDM4A is independent of activation of the NF-κB and HIF pathways, therefore suggesting an alternative mechanism for regulation of macrophage activation and function. As oxLDL serves as a major risk factor and a core microenvironmental component of atherosclerosis, the present findings might be particularly implicated in atherosclerotic diseases. Thus, they might provide a new insight into the mechanism underlying uncontrolled inflammation that drives atherosclerotic pathogenesis. In this context, they also suggest that KDM4A might represent a novel target for epigenetic therapy in the prevention and treatment of atherosclerotic diseases, particularly their fatal complications such as heart attack and ischemic stroke. In addition, it is worth to mention that the present study provides the first piece of evidence for a novel function of KDM4A in regulation of macrophage activation and function. Given the lack of understanding of its role in inflammation, KDM4A also warrants further attention in other inflammatory or immune diseases, as well as tumor immunity.

## MATERIALS AND METHODS

### Cells and reagents

Murine macrophage cell line RAW264.7 [23] and human monocytic cell line THP-1 were obtained from American Type Culture Collection (ATCC; Rockville, MD). RAW264.7 cells were maintained in DMEM medium (Corning, NY, USA) containing 10% heat inactivated fetal bovine serum (FBS; Gibco, Thermo Fisher Scientific, Frederick, MD) and supplemented with 2 mM L-glutamine. THP-1 cells were cultured in RPMI-1640 medium (Corning) containing 10% heat inactivated FBS. To prepare human macrophages (M0), THP-1 cells were incubated with 30 ng/ml phorbol 12-myristate 13-acetate (PMA; Sigma, St. Louis, MO) for 48 hrs, followed by incubation with RPMI medium without PMA for additional 24 hrs [28].

Human oxidized LDL was obtained from Peking Union-Biology Co. Ltd (Beijing, China). The IκB kinase (IKK) inhibitor parthenolide [36] and the pan-selective Jumonji histone demethylase inhibitor JIB-04 [35] were purchased from Merck Millipore (Billerica, MA).

### Plasmids and lentiviral transduction

Constructs encoding short hairpin RNA (shRNA) specifically targeting KDM4A was prepared using the pGreenpuro vector flagged with green fluorescent protein (GFP). Two shRNAs target different DNA sequences of the KDM4A gene (shKDM4A-1: CGTTGATGAGTGGAATATT and GCTGAAGAATGTCAAACTA; shKDM4A-2: CGAACA TCCTACGACGATATTCTC and AGCACGTTGATGA GTGGAATACTC). Control shRNA targets a scrambled sequence.

For lentiviral packing, HEK 293T cells were seeded in complete DMEM medium one day before transfection. Cells were then transfected with constructs encoding shKDM4A-1, shKDM4A-2, or control scramble shRNA along with psPAX2 and PMD2G using Lipofectamine 2000 (Invitrogen, Carlsbad, CA). After 6 hrs, transfection medium was replaced with fresh DMEM medium. Viral supernatants were harvested at 24 and 48 hrs post-transfection.

For lentiviral infection, RAW264.7 cells were cultured with conditional medium containing viral particles for 48 hrs. Transduction efficiency achieved more than 60–70% determined by monitoring the percentage of GFP-positive cells. Subsequently, transducted RAW264.7 cells were then selected by puromycin. Western blot analysis was performed to monitor expression of KDM4A.

### Western blot analysis

After washed with ice-cold PBS, whole cell lysates were prepared in RIPA lysis buffer (Cell Signalling Technology, Danvers, MA) containing phenylmethylsulphonyl fluoride (PMSF) and phosphatase inhibitors (Roche, Berlin, Germany). Total protein was quantified by using BCA protein assay (Thermo Fisher Scientific). Equal amounts of protein (20 or 30 μg) were resolved by 8% sodium dodecyl sulfate polyacrylamide gel electrophoresis (SDS-PAGE), followed by transferring to polyvinylidene fluoride (PVDF) membranes. Membranes were blocked with PBS-Tween 20 containing 5% bovine serum albumin (BSA) at room temperature for 1 hr and then probed with the appropriate dilution of primary antibody overnight at 4°C, followed by incubation with a 1:5000 dilution of horseradish peroxidase-conjugated secondary antibody (Dingguo, Beijing, China) at room temperature for 1 hr. After washing twice in PBS-Tween 20, the proteins were visualized by using an enhanced chemiluminescence (ECL) kit (PerkinElmer, Waltham, MA). The following antibodies were used as primary antibodies: anti-HIF-1β/ARNT (rabbit), anti-HIF-1α (rabbit), anti-Arg1 (rabbit), anti-phospho-NF-κB p65 (Ser536; rabbit), and anti-iNOS antibodies (rabbit) from Cell Signalling Technology; anti-KDM4A antibody (mouse) from Santa Cruz Biotechnology (Dallas, TX). Where indicated, the blots were reprobed with β-actin antibody (rabbit, Cell Signalling Technology) to ensure equal loading and transfer of proteins.

### Qualitative real-time PCR (qPCR)

Total RNA was extracted from cells using an Easy Pure RNA kit (Transgene Biotech, Beijing, China) as per manufacturer’s instruction. 1 μg per condition of total RNA was reversely transcribed to cDNA, which was amplified with SYBR (Roche) by real-time PCR as follows: 95°C for 10 minutes, followed by 40 cycles of 95°C for 10 seconds, and then 60°C for 30 seconds. All PCR reactions were run in triplicate, and gene expression relative to GAPDH was calculated using the 2^−ΔΔCT^ method.

Primers for mouse genes include: *Tnf-α*: forward, 5′-TCGTAGCAAACCACCAAGT G-3′; reverse, 5′-GG AGTAGACAAGGTACAACCCA-3′. *IL-1β*: forward, 5′-GAAGTTGAC GGACCCCAAAA-3′; reverse, 5′-CCA CAGCCACAATGAGTGATAC-3′. *Mcp1*: forward, 5′-GCT CAGCCAGATGCAGTTA-3′; reverse, 5′-CTGCTGGT GATCCTCTTGTAG-3′. *iNos*: forward, 5′-GTTCTCAG GCCAACAATACAAGA-3′; reverse, 5′-GTGGACGGG TCGATGT CAC-3′. *Arg1*: forward, 5′-ACCTGGCCTTT GTTGATGTC-3′; reverse, 5′-ACTGCCAGA CTGT GGTCTCC-3′. *Vegf*: forward, 5′-ACTGGACCCTGG CTTTACTG-3′; reverse, 5′-TCT GCTCTCCTTCTGTC GTG-3′. *KDM4A:* forward, 5′-ACCCCAGTGCTCGG ATCAT-3′; reverse, 5′-GGAGGAACGACCTTGGCTA-3′. *Gapdh*: forward, 5′-TGCACCACCAACTGC TTAGC-3′; reverse, 5′-GGATGCAGGGATGATGTTCT-3′.

Primers for human genes include: *iNos*: forward, 5′-CCCCAGCCTCAAGTCTTATTT C-3′; reverse, 5′-GC AAGTTCCATCTTTCACCCAC-3′. *KDM4A:* forward, 5′-GAAGCCA CGAGCATCCTATGA-3′; reverse, 5′-GC GGAACTCTCGAACAGTCA-3′. *Tnf-α*: forward, 5′-ATC CTGGGGGACCCAATGTA-3′; reverse, 5′-AAAAGAA GGCACAGAGGCCA-3′. *Vegf*: forward, 5′-CCACGA CAGAAGGAGAGCAGAAGTCC-3′; reverse, 5′-CGTTA CAGC AGCCTGCACAGCG-3′. *Gapdh*: forward, 5′-AGA AGGCTGGGGCTCATTTG-3′; reverse, 5′-AGGGGCC ATCCACAGTCTTC-3′.

### Cytometric Bead Array (CBA) and ELISA analyses of soluble cytokines

After cells were incubated with oxLDL for 24 hrs, culture media were collected to measure the protein levels of soluble cytokines. The concentrations of TNF-α and MCP-1 were determined by LSRFortessa flow cytometry (BD Biosciences, San Jose, CA) using a Cytometric Bead Array kit (BD PharMingen, Franklin Lakes, NJ) as per manufacturer’s instruction. Individual sample (50 μl/each) was tested in duplicate. The concentrations of cytokines were quantified by CellQuest Pro using a CBA software. The concentration of IL-1β was measured by ELISA using a mouse IL-1β ELISA kit (R&D System, Minneapolis, MN) as per manufacturer’s instruction.

### Analyses of cell viability and apoptosis

Cell viability was evaluated using a Cell Counting Kit-8 (CCK8) kit (Dojindo Laboratories, Kumamoto, Japan) as per manufacturer’s instruction. The percentage of apoptosis was determined by flow cytometry using Annexin V-PE and 7AAD (BD Biosciences, San Diego, CA) double staining.

### Statistical analysis

The values represent the means ± standard deviation (SD) for at least three independent experiments performed in triplicate. The significance of differences between experimental conditions was determined using two-tailed Student’s *t*-test. All statistical analyses were conducted using GraphPad Prism version 5. *P* < 0.05 is considered as statistically significant.

## SUPPLEMENTARY MATERIALS FIGURES AND TABLES


