# Supplementary material for: Identification of the histone lysine demethylase KDM4A/JMJD2A as a novel epigenetic target in M1 macrophage polarization induced by oxidized LDL
Source: Oncotarget. 2017 May 10;8(70):114442–56. doi: 10.18632/oncotarget.17748 (PMC5777704; doi:10.18632/oncotarget.17748)
Supplement: Supplementary file 1 [file oncotarget-08-114442-s001.pdf]

Identification of the histone lysine demethylase KDM4A/JMJD2A as a novel epigenetic target in M1 macrophage polarization induced by oxidized LDL

Supplementary Materials

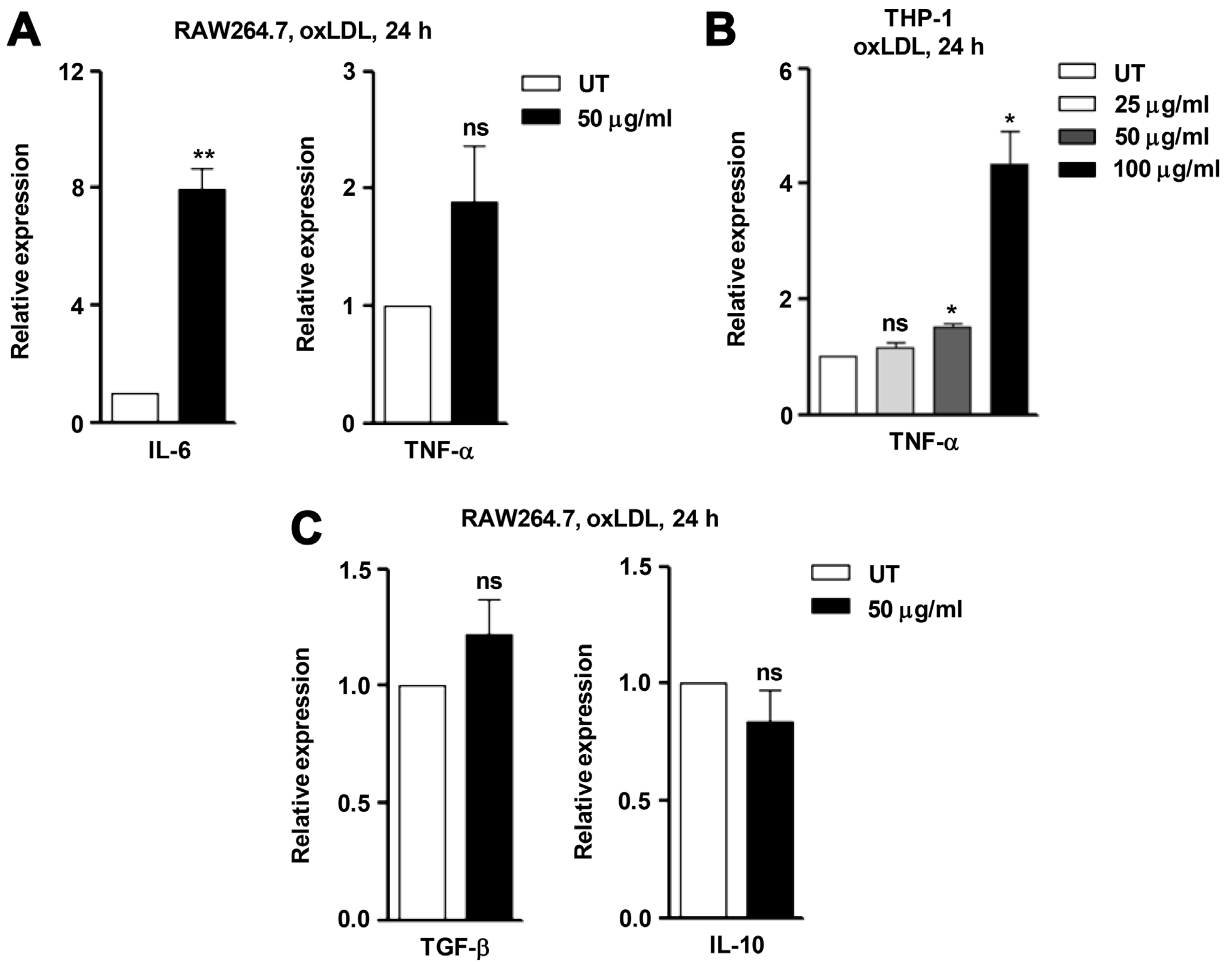

Supplementary Figure 1: OxLDL promotes expression of the M1 inflammatory genes in macrophages. (**A**) RAW264.7 cells were stimulated with oxLDL at 50 μg/ml for 24 hrs, the mRNA levels of the M1 genes IL-6 and IFN-γ were determined by qPCR. (**B**) THP-1-derived human macrophages were exposed to oxLDL at 25, 50, 100 μg/ml for 24 hrs, after which mRNA expression of TNF-α was monitored by qPCR. (**C**) RAW264.7 cells were treated as described in panel 1A, the mRNA levels of the M2 genes TGF-β and IL-10 were determined by qPCR. Values represent the means ± SD for at least three independent experiments performed in triplicate. $^{*}P < 0.01$, $^{**}P < 0.05$, compared to untreated control (UT).

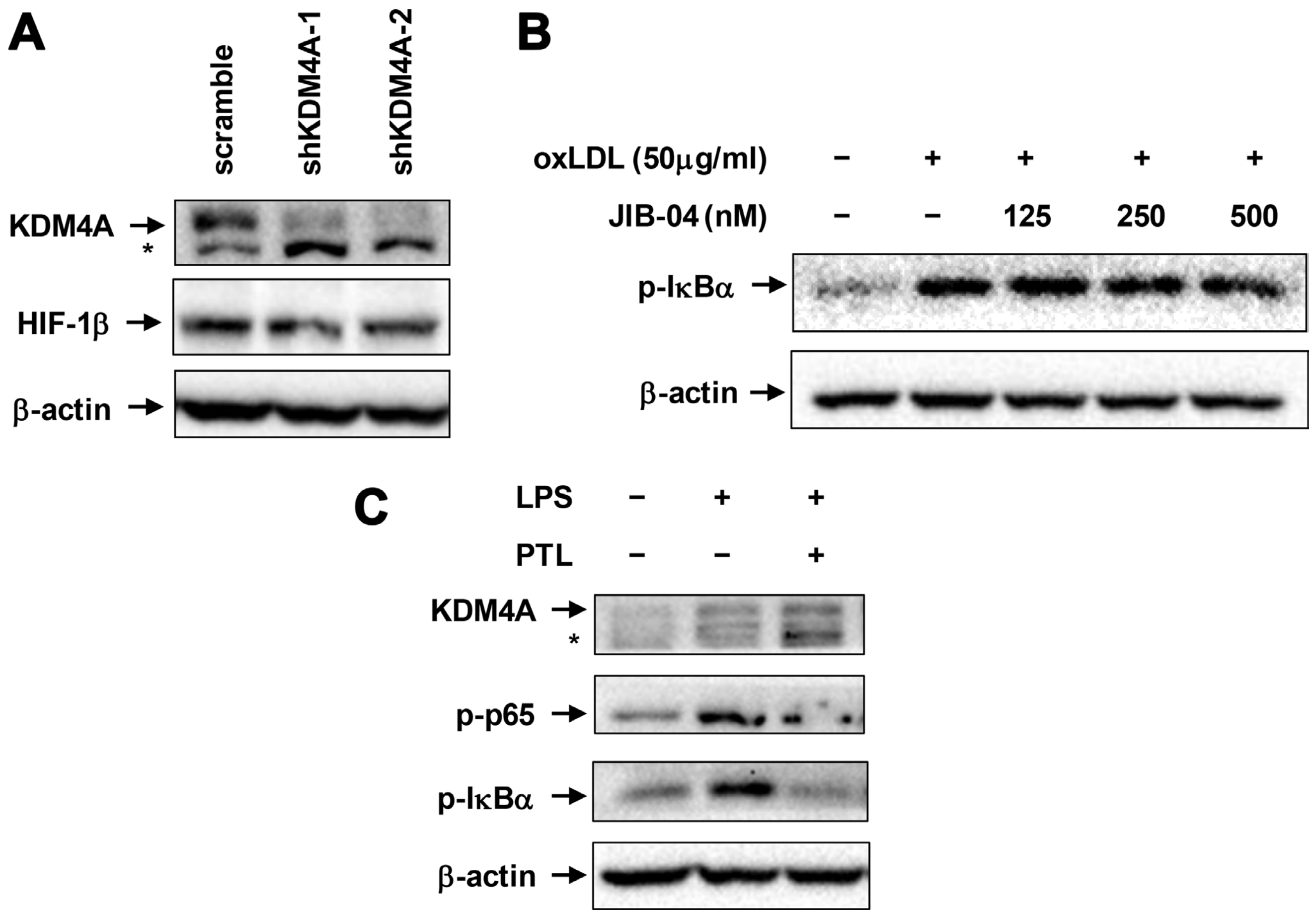

Supplementary Figure 2: KDM4A up-regulation is independent of NF-κB and HIF activation in RAW264.7 cell exposed to oxLDL. (**A**) RAW264.7 cells transfected with shKDM4A-1 and -2 as well as scramble control shRNA were subjected to Western blot analysis for expression of KDM4A and HIF-1β. (**B**) RAW264.7 cells were exposed for 1 hr to 50 μg/ml oxLDL with or without pre-treatment with the JMJD inhibitor JIB-04 (125–500 nM) for 4 hrs, after which S32/S36 phosphorylation of IκBα, a direct downstream target of IKKβ, was monitored by Western blot analysis. (**C**) RAW264.7 cells were exposed for 2 hrs to 1 μg/ml lipopolysaccharide (LPS), an agent known to induce M1 polarization via NF-κB activation, with or without pre-treatment with the IKK inhibitor parthenolide (PTL) for 2 hrs, after which expression of phosphorylated p65 (S536) and IκBα (S32/S36) as well as KDM4A was monitored by Western blot analysis. * undefined band.
